# Inflammatory pattern of the infrapatellar fat pad in dogs with canine cruciate ligament disease

**DOI:** 10.1186/s12917-018-1488-y

**Published:** 2018-05-16

**Authors:** Manuel R. Schmidli, Bettina Fuhrer, Nadine Kurt, David Senn, Michaela Drögemüller, Ulrich Rytz, David E. Spreng, Simone Forterre

**Affiliations:** 1 Division of Small Animal Surgery and Orthopaedics, Department of clinical veterinary medicine, Vetsuisse Faculty, University of Bern, Länggassstrasse 128, 3012 Bern, Switzerland; 2 Animal Clinic Thun Süd, Thun, Switzerland; 3 Institute of Genetics, Department of Clinical Research and Veterinary Public Health, Vetsuisse Faculty, University of Bern, 3001 Bern, Switzerland

**Keywords:** Infrapatellar fat pad, Inflammatory pattern, Cranial cruciate ligament disease, CCLD, Dogs, Osteoarthritis

## Abstract

**Background:**

Despite the importance of inflammation during the pathogenesis of cranial cruciate ligament disease (CCLD) in dogs and despite the latest knowledge suggesting a significant role of adipose tissue in osteoarthritis, the infrapatellar fat pad (IFP) was up to now mostly disregarded in veterinary investigations. In the present study, the inflammatory activity of the IFP, the main adipose structure within the stifle joint, was thoroughly investigated to evaluate its potential impact in the pathogenesis of this common disease of our canine companions. Samples of IFP, subcutaneous adipose tissue (ScAT) of the thigh and synovial fluid in both diseased (*n* = 36) and healthy control (*n* = 23) dogs were tested for their immune cell composition but also for interleukins (IL-1β, IL-6, IL-8, IL-10), degradative enzymes (MMP-1, MMP-3, MMP-13, TIMP-2, iNOS) and adipokines (leptin and adiponectin). Characterization of the immune cell composition was ascertained by fluorescence activated cell sorting. Gene expression and protein release of the inflammatory markers was determined by real RT-qPCR and ELISA.

**Results:**

IFPs of dogs with CCLD had a significantly increased immune cell count with T cells (CD3) as the most abundant immune cells. T cells and macrophages (CD14) were significantly increased compared to healthy controls or corresponding ScAT. In addition, IFPs of dogs with CCLD demonstrated a significant increase on gene as well as protein level of multiple inflammatory indicators (IL-1β, IL-6, MMP-1, MMP-13) compared to the other tissues. TNFα was only increased on gene expression. Adipokine analysis showed higher secretion of adiponectin and lower leptin secretion in IFP from dogs with CCLD than from controls. In the synovial fluid from dogs with CCLD concentrations of IL-1β, MMP-1, MMP-13 as well as leptin were significantly increased compared to the synovial fluid from healthy control dogs.

**Conclusions:**

The present study indicates that the IFP is a potential contributory factor in the pathogenesis of CCLD, due to its inflammatory phenotype and the proximity within the stifle joint. To determine the extent of this possible inter-relationship, further studies need to be undertaken.

## Background

Cranial cruciate ligament disease (CCLD) is a common disorder and the most common reason for hind limb lameness in canine patients [1, 2]. The majority of veterinary clinicians employ surgical intervention to manage CCLD, yet no such procedures have been shown to prevent further progression of osteoarthritis (OA) [3, 4]. Consequently, a better understanding of the pathogenesis of osteoarthritic changes seen during CCLD would be advantageous in order to facilitate the development of more effective therapeutic strategies. Besides contributing factors like age, body condition score (BCS), stifle morphology and ligament matrix composition, there is clear evidence that inflammation plays a prominent role in the progression of OA during CCLD [5, 6]. Dogs with CCLD typically have inflammatory changes in the synovial intima, the epiligament and the core region of the cruciate ligament [7, 8]. Moreover, various pro-inflammatory and degrading factors known to be involved in the initiation and progression of OA secondary to CCLD are measurable in synovial fluid [9–11]. The ligament, synovium and articular cartilage are regarded as the principal sources of factors secreted into synovial fluid during CCLD. Pro-inflammatory cytokines such as interleukin-1 (IL-1), IL-6, IL-8 and tumor necrosis factor (TNF)-α have been found to be synthesized by synovium [12, 13]. Matrix metalloproteinases (MMP) from CCL are implicated in the initiation of OA when ligament tissue is exposed to the joint environment [13, 14]. However, the relative contribution of each compartment of the joint to the pathophysiology remains unclear, as is the length of exposure time required in order to initiate the disease.

A characteristic part of the stifle joint, the infrapatellar fat pad (IFP), has mostly so far been disregarded in veterinary medicine. The IFP was considered to have merely biomechanical functions within the knee, serving primarily as a shock absorbing structure and to improve the distribution of lubricant [15]. Due to its intraarticular but extrasynovial localization and the now recognized potential of adipose tissue to act as a source of inflammatory factors, the IFP is progressively gaining attention related to OA, mainly in human medicine [16–19]. New concepts postulate that joint overload is not the sole factor that may result in the development of OA; inflammatory responses within adipose tissue can also play a role. The fact that obese people have an increased risk of developing not only OA of the knee but also of the hand supports this hypothesis. Besides adipocytes, adipose tissue also contains fibroblasts, endothelial cells and immune cells [20–22]. Adipocytes are capable of producing a vast spectrum of inflammatory mediators and potentially contribute to OA [23, 24]. Several studies demonstrated an infiltration of macrophages, lymphocytes and granulocytes in the human IFP during OA [19, 25]. It is postulated that immune cells interact with adipocytes and together are responsible for the release of several cytokines like adipokines or interleukins [26]. Despite increasing effort in research into this field, so far little knowledge is available on how the canine IFP influences both the production and the release of inflammatory mediators.

The aim of our project was to identify the level of inflammatory activity in canine IFP during CCLD. Therefore, a characterization of the immune cell composite, the gene activity on mRNA basis, as well as the final protein production of the important inflammatory mediators in this context was required. To estimate its inflammatory potential those parameters were measured within the IFP, subcutaneous adipose tissue (ScAT) and the synovial fluid of both diseased and healthy control dogs. Our hypothesis was, that the IFP during CCLD shows a significantly increased inflammatory pattern compared to IFP of healthy control dogs and compared to corresponding ScAT.

## Methods

### Dogs

A total of 59 dogs were enrolled in the present study. Thirty-six client-owned dogs were classified as the CCLD group. The presurgical diagnosis was made based on physical examination; lameness in the affected hind limb that was localized to the stifle joint, detection of cranial drawer, stifle joint effusion; and radiographic evidence of new bone formation in the stifle joint. Study inclusion criteria for the CCLD group were a diagnosis of a complete or partial CCL rupture confirmed by probing and observation during arthroscopy or arthrotomy and unremarkable results of routine hematologic and serum biochemical analyses. Clinical examination of all cases was undertaken and cases presenting any clinical signs indicating illness other than CCLD were excluded, as were those exhibiting traumatic CCL rupture or with a history of performed intra-articular injections. Dogs were admitted for surgical treatment either to the Animal Clinic Thun Süd or the Small Animal Clinic at the Vetsuisse Faculty, University of Bern. A standard anaesthesia protocol was followed in all dogs. This included intramuscular premedication with acepromazine (0.03 mg/kg) and methadone (0.2 mg/kg), induction with propofol (2–8 mg/kg intravenously to effect), and maintenance with isoflurane in an air-oxygen mixture, delivered using a rebreathing system (end-tidal concentration, 1.2–2%). An ultrasound-guided femoral and sciatic nerve block was performed with ropivacaine (0.3 mL/kg 0.5%) for each nerve. A control group consisted of 23 healthy dogs. Inclusion into the control group necessitated normal results for routine hematologic and serum biochemical analyses along with absence of any history of orthopaedic disease, particularly evidence of stifle OA and/or CCLD confirmed during sample collection. Additionally, control dogs had not received any medication for one month prior to sampling. Twenty-one control dogs were euthanized at the end of an unrelated experimental study (BS2197) with an overdose of pentobarbital intravenously after induction of general anaesthesia in accordance with the AVMA guidelines for the euthanasia of animals. Two dogs from the control group were involved in a road traffic accident and were euthanized due to poor prognosis. Various parameters were recorded for each dog including breed, gender, age, and weight. Concerning the BCS, each dog was classified by one individual using a nine-point scale [27] which was sub-grouped into three categories, namely lean to ideal (3–5), overweight (6–7) and obese (8–9). Additionally, the medication history during the last 6 months prior to surgical treatment and the duration of lameness were recorded. Gross pathologic features were assigned to four joint alterations (synovitis, development of osteophytes, meniscus infringement and articular cartilage lesions) as indicated in Table 1. All procedures and the written owner consent form were approved by the commission of animal experimentation of the Canton of Berne and Basel, Switzerland (BE116/13; BS2197). Prior to study enrolment, written owner consent was obtained for each dog.

**Table 1:** Morphological grading system [60, 61]

Grade				
Structure	0 (normal)	1 (mild)	2 (moderate)	3 (severe)
Synovitis^a^	Thin synovial membrane with thin scattered vessels	Isolated thickening of synovial membrane with granulations and small vessels	Synovial membrane with abundant villous projections and large vessels	Synovial membrane with large villi and abundant vasculature
Osteophytes^b^	none	solitary	multiple	confluent
Medial meniscus	normal	Fibrillated surface	Undisplaced tear	Displaced tear
Articular cartilage lesions^c^	none	Solitary fibrillation	Multiple areas of fibrillation up to solitary erosion	Multiple erosions to the level of subchondral bone

^a^Arthroscopic evaluation of the synovial membrane

^b^Development of osteophytes along the femoral condyles, the dorsal aspect of the patellar sulcus and within the femoral notch itself, evaluated via arthroscopy

^c^Area of femoral trochlea visualized via arthroscopy

### Sample collection

On the day of surgical intervention for CCL rupture, each dog was anaesthetized and stifle joints were aseptically prepared. A synovial fluid sample (0.1 to 0.4 mL) was aseptically collected by percutaneous arthrocentesis. Tissue samples from the thigh ScAT, removed next to the skin incision and the IFP (wet weight, each 1 to 5 g) were obtained from dogs during arthroscopy or arthrotomy. Samples from control dogs were harvested within 30 min after euthanasia. Depending on the conducted experiment, tissue samples were portioned and either immediately processed in cold Dulbecco’s modified eagle medium (DMEM, Thermo Fisher Scientific, Zug, Switzerland) for flow cytometry or ELISA procedures, or submerged in RNAlater (Qiagen, Hilden, Germany) for quantitative RT PCR. All samples were stored at − 80 °C until further analysis unless otherwise stated. Owing to the limited size of the samples, not all experiments could be performed with each sample.

### Cell isolation of IFP and ScAT

Freshly obtained adipose tissue samples were dissociated by being cut into small pieces. Stromal vascular cells were isolated from 1 g minced tissues using collagenase (C6885, Sigma-Aldrich, Buchs, Switzerland; 1.3 mg/ mL DMEM/ g adipose tissue) in DMEM on a shaker at 37 °C. After the one-hour digestion, the cell suspension was filtered through a 70-μm-mesh (BD Biosciences, Allschwil, Switzerland) in order to remove undigested tissue. The filtered cell suspension was centrifuged at 300 g for 10 min. The floating adipocyte fraction including the supernatant was collected and centrifuged again for 10 min at 300 g. After the second spin, the two pellets were combined and resuspended into 10 mL of 4 °C cold FACS buffer (PBS containing 0.5% bovine serum albumin and 2 mM EDTA). Following an additional washing step, cells were counted and then diluted to a final concentration of 1 × 10^7^ cells/ mL. Care was taken during specimen handling to ensure that cell isolation was as consistent as possible. As much tissue as possible was analyzed to maximize the number of cells isolated; however, in some individual samples, the number of cells isolated imposed limitations on the number of CD makers that could be studied with flow cytometry.

### Flow cytometry

An aliquot of 1 × 10^6^ cells was resuspended in 100 μL of FACS buffer before staining with primary antibodies as indicated in Table 2. Before staining with MAC387, samples were permeabilized and fixed using 1% paraformaldehyde for 10 min, followed by 1% Triton for 20 min. All aliquots were incubated with a single antibody against CD3, CD14, CD19, CD21 and MAC387 for 50 min on ice. A second antibody (dilution 1: 800; goat anti-mouse IgG DyLight 649 or 488, AbD Serotec, Germany) was used for CD3 and MAC387 for an additional 30 min. Following staining procedures, cells were washed twice and then resuspended in 0.5 mL FACS buffer. Subsequently, antibody binding was visualized and compensation procedures were performed using a LSRII BD Bioscience flow cytometer. Control gates were set based on staining with isotype controls. Side light-scatter versus forward light-scatter characteristics were used to identify neutrophils, monocytes and lymphocytes. For data acquisition the same instrument settings were used for each sample and 50,000 events were recorded to generate frequency determination per antibody. Frequency of cell subsets was determined as a percentage of total cells operated by Diva 6 software (BD).

**Table 2:** Primary antibodies for IFP and ScAT staining

Antibody	Clone	Dilution per 10^6^ cells	Supplier
Mouse anti-canine CD3	CA17.2A12	1:10	AbD Serotec
Mouse anti-human CD14-Alexa 488	M5E2	1:20	BD Biosciences
Mouse anti-macrophage MAC387	MAC387	1:100	abcam^a^
Mouse anti-CD19-FITC	MB19–1	1:50	abcam^a^
Mouse anti-canine CD21-Alexa 647	CA2-1D6	1:10	AbD Serotec

^a^abcam, Cambridge, UK

### RNA extraction

Total RNA from IFP and ScAT was extracted using a combination of phenol/guanidine hydrochloride reagent, RNeasy purification and on-column DNase treatment (Qiagen) following a protocol for fatty tissue (Qiagen). Briefly, 100 mg of tissue was homogenized in 1 mL TRIzol® (Thermo Fisher Scientific) with a Qiagen TissueLyser for 3 min. After extraction with 200 μL of chloroform and ethanol precipitation, the sample was loaded on the RNeasy spin column. An on-column DNase (Qiagen) treatment was included. Total RNA was finally eluted in 50 μL of RNAse-free water and stored at − 80 °C until use. Quantity and quality of total RNA were assessed by using an Agilent 2100 bioanalyzer (Santa Clara, USA) for calculation of the concentration, the ratios 18S/28S and RNA integrity number.

### cDNA synthesis

Reverse transcription was performed using SuperScript IV reverse transcriptase (Invitrogen, Thermo Fisher Scientific) in accordance to the manufacturer’s instructions with few changes. Briefly, 2–5 μg (11 μL) template RNA was preincubated with 50 μM (1 μL) oligo-d(T)20 and 10 mM (1 μL) dNTP mix at 65 °C for 5 min. After incubation on ice, 4 μL 5 x first strand buffer (containing 250 mM Tris-HCl, pH 8.3, 375 mM KCl, 15 mM MgCl2), 1 μL of 0.1 M DTT, 1.5 μL nuclease-free water and 0.5 μL Superscript IV reverse transcriptase were added to each sample and the combined reaction mixture was incubated at 50 °C for 30 min. Reverse transcriptase activity was terminated by incubation at 65 °C for 15 min, and samples were stored at − 80 °C until use.

### Quantitative real-time reverse transcriptase PCR (RT-qPCR) assay

IFP and ScAT from both groups were evaluated for gene expression of matrix molecule proteinases (MMP-1, MMP-3, MMP-13), inhibitor TIMP-2, inflammatory indicators (IL-1β, IL-6, IL-8, IL-10, iNOS, TNFα) and a housekeeping gene (β-actin). Canine-specific transcript sequences for selected factors were designed based on gene bank information and synthesized by Microsynth (Balgach, Switzerland) as presented in Table 3. Quantitative real-time reverse transcriptase PCR was performed in duplicates using the 7300 Real Time PCR System (Applied Biosystems) in a 96-well format, with three no template controls used for each assay. The following protocol was executed for each transcript sequence: The reaction volume in each well consisted of 10 μL Power SYBR® green (Applied Biosystems), 0.8 μL of 10 μM forward primer, 0.8 μL of 10 μM reverse primer, 7.4 μL of nuclease-free water and 1 μL of sample cDNA. The standard amplification condition consisted of 1 cycle at 95 °C for 5 min, followed by 42 cycles of 95 °C for 10 s and 60 °C for 30 s and extension at 72 °C for 30 s. Subsequently, a terminal cycle of 95 °C for 15 s, 60 °C for 30 s and finally 95 °C for 15 s was performed. Fluorescence was detected during the extension step of each cycle and during the melt curve analysis for SYBR^®^ green Cycler (Applied Biosystem). Real-time data was then analyzed by using the 7300 System Software (Sequence Detection Software, version 1.3). Data processing was conducted using the 2-ΔΔCT method [28]. The data then was finally presented as the fold change in gene expression normalized to the internal control gene β-actin and relative to the ScAT of healthy controls.

**Table 3:** Oliconucleotide primers used for RT-qPCR

Target gene	Forward (F) and reverse (R) primer (5′-3′)	Amplicon size (bp)	Annealing temperature (°C)
IL-1β	F-GCCAAGACCTGAACCACAGT	96	60.16
	R-CTGACACGAAATGCCTCAGA		59.98
IL-6	F-ACCGGTCTTGTGGAGTTTCA	102	60.55
	R-CAGGATCTTGGTACTCATGTGC		59.62
IL-8	F-TTCAGAACTTCGATGCCAGT	90	58.44
	R-GGGCCACTGTCAATCACTCT		60.12
IL-10	F-CAGAGCACCCTACCTGAGGA	97	60.40
	R-AAGTCTTCACCCTCCCGAAG		60.62
TNFα	F-GAGCACTGAAAGCATGATCC	108	58.39
	R-GAGAAGAGGCTGAGGCAGAA		59.83
iNOS	F-GTCTGGGAGCCATCATGAAC	98	60.48
	R-GACAAATTCAATGGCTTGAGG		59.56
MMP-1	F-TGAATTGGGTCATTCTCTTGG	98	59.92
	R-CCTGAGATAGCTGGACATTGC		59.85
MMP-3	F-CCTAGCGCTCTGATGTACCC	90	59.86
	R-GGACTGGATGCCATTCACAT		60.76
MMP-13	F-AGTTCGGCCACTCCTTAGGT	102	60.13
	R-CATCGGGAAGCATAAAGTGG		60.46
TIMP-2	F-TGTTCAAAGGACCAGACAAGG	109	60.13
	R-TTCTTTCCTCCGATGTCCAG		60.19
β-actin	F-GGACCTGACCGACTACCTCA	91	60.11
	R-CTTGATGTCACGCACGATTT		59.72

### Quantification of selected cytokines, MMPs and adipokines by ELISA

For ELISA, tissue explants (100 mg/ mL) from freshly isolated and washed IFP and ScAT were cultured in DMEM for 48 h at 37 °C and 5% CO_2_. To evaluate spontaneous secretory activities of IFP and ScAT, the concentration of selected factors was measured in triplicates in culture supernatants of the tissue explants using commercially available ELISA kits, including canine IL-1β (SEA563Ca, Cloud-Clone Corp, TX, USA), canine IL-6 (SEA079Ca, Cloud-Clone), canine MMP-1 (CSB-E14057c, Cusabio Biotech Co, China), canine MMP-3 (CSB-E13621c, Cusabio), canine MMP-13 (CSB-E14055c, Cusabio), dog adiponectin (CY-8052, CircuLex, Nagano, Japan) and canine leptin (EZCL-31 K, Millipore Corporation, MA, USA). Moreover, the concentration of these factors was also quantified in synovial fluid. The assays were performed according to the manufacturer’s protocols. Sample concentrations were calculated based on standard dose-response curves. Results are demonstrated as cytokine content per 100 mg tissue/ mL.

### Statistics

All statistical analyses were carried out using MedCalc version 17.8 (MedCalc Software bvba, Ostend, Belgium; http://www.medcalc.org; 2017). Summary statistics were performed and normality assessed using the D’Agostino-Pearson test. Data was reported as mean (range) and 95% CI or median and 95% CI for data that was not parametrically distributed. As some data were normally distributed but others were not, nonparametric statistics were used. Fisher’s exact test was used to assess differences in age and BCS between the two groups. Differences in cell phenotype, gene expression and protein secretion between the two groups were evaluated for ScAT, IFP and synovial fluid using a Mann-Whitney test. Variations of same parameters within the groups were calculated using a Wilcoxon signed-rank test. Associations between analyzed inflammatory parameters and gross synovitis scores, grade of CCL rupture as well as clustered BCS were evaluated using Kruskal-Wallis tests. A Spearman’s rank correlation was used to examine the relationship between the different inflammatory parameters and duration of lameness. All assessments were two-tailed and significance was set at *p* < 0.05.

## Results

### Dogs

#### Cranial cruciate ligament diseased group

The CCLD group consisted of 21 females (8 intact, 13 spayed) and 15 males (3 intact, 12 castrated) which represented the following breeds: Labrador Retriever (*n* = 10), Golden Retriever (*n* = 5), Border Collie (*n* = 4), with remaining breeds represented by 3 or fewer dogs. Mean (range) patient age and body weight were 5.9 years (range: 1.2–10.9) and 36.6 kg (range: 14.5–76.0). Relating to the BCS, 19 dogs were in the lean to ideal category, 10 dogs were grouped as overweight, and seven dogs were obese. Within one month prior to surgery, 14 dogs had received daily, 11 dogs intermittent, and 11 dogs no medication (carprofen, firocoxib, meloxicam, robenacoxib, or prednisone), respectively. A partial CCL rupture was diagnosed in 15 dogs (four left, 11 right) and a complete rupture in 21 dogs with 14 on the left and seven on the right stifle joint. Sixteen dogs (44%) suffered a rupture of the contralateral CCL within a mean time of 13 months (range: 2–48 months) after the diagnosis of unilateral CCL rupture. The mean duration of lameness was 96 days (range: 7–360 days). Based on the grading of the morphological alteration (Table 1) within the joint, five dogs showed mild, 14 dogs moderate and 17 dogs severe synovitis. Dogs with a complete CCL rupture showed statistically significant higher scores of synovitis (*p* = 0.038). Development of osteophytes was observed in 23 dogs, 13 dogs had solitary and 10 dogs multiple osteophytes. Meniscal injury was noted in 16 stifles, whereupon a fibrillated surface was detected in five dogs and 11 dogs had a displaced tear, however, there was no correlation with the degree of synovitis (rs = 0.31; *p* = 0.073; 95% CI -0.03-0.58).

#### Control

The control group comprised 20 Beagles, two Labrador Retrievers and one Hovawart that fulfilled the inclusion criteria. Of these control dogs, eight were castrated males and 15 spayed females. Mean age and weight were 4.1 years (range: 1.1–10.8 years) and 13.2 kg (range: 7.4–33.2 kg). Based on the BCS, 16 dogs were classified as lean to ideal and 7 dogs as overweight. In all control dogs, morphological features of joint structures appeared normal.

### Inflammatory pattern of IFP, ScAT and synovial fluid

#### Immune composition of the IFP and ScAT

**Fig. 1:**
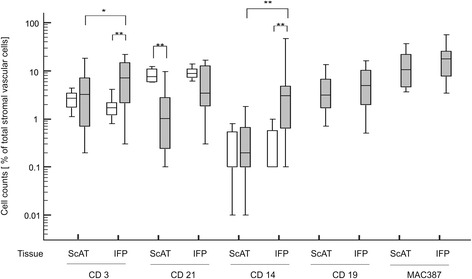
Immune cell composition of the ScAT and IFP. Cells were isolated from the stromal vascular fraction of ScAT and IFP of control dogs (white boxes) and CCL diseased dogs (grey boxes). Immune cells were characterized by flow cytometry and shown as percentage of total stromal vascular cells. * denotes statistically significant differences with *p* < 0.005, ** *p* < 0.0005

Immune cell composition of IFP and paired ScAT from diseased and control dogs are presented in Fig. 1. Flow cytometry analysis demonstrated a significant difference with more cells/ g tissue present in the IFP of dogs with CCLD than in the paired ScAT. T cells (CD3) were the most abundant immune cells in the IFP from diseased dogs followed by B cells (CD19/21) and macrophages (CD14) in similar percentages. Beside macrophages, neutrophils were numerously recruited in the IFP stained with the antibody MAC387. Moreover, a statistically significant difference was found for T cells (*p* < 0.005) and macrophages (*p* < 0.0005), with elevated levels in the IFP compared to the paired ScAT of dogs with CCLD and to both control adipose tissues from healthy control dogs. In control dogs, the most abundant cells were B cells with no difference between IFP and ScAT. The level of B cells found in ScAT from diseased dogs was significantly lower (*p* < 0.0005) in comparison to that from control dogs. No relationship was seen between number of CD3^+^ or CD14^+^ cells and duration of lameness (CD3, rs = 0.07, *p* = 0.7; 95% CI -0.4-0. 5; CD14, rs = − 0.38, *p* = 0.08; 95% CI -0.7-0.05), grade of synovitis (CD3, *p* = 0.4; CD14, *p* = 0.92), medication (CD3, *p* = 0.37; CD14, *p* = 0.9) or BCS (CD3, *p* = 0.10; CD14, *p* = 0.19).

### Inflammatory gene expression in IFP and ScAT

**Fig. 2:**
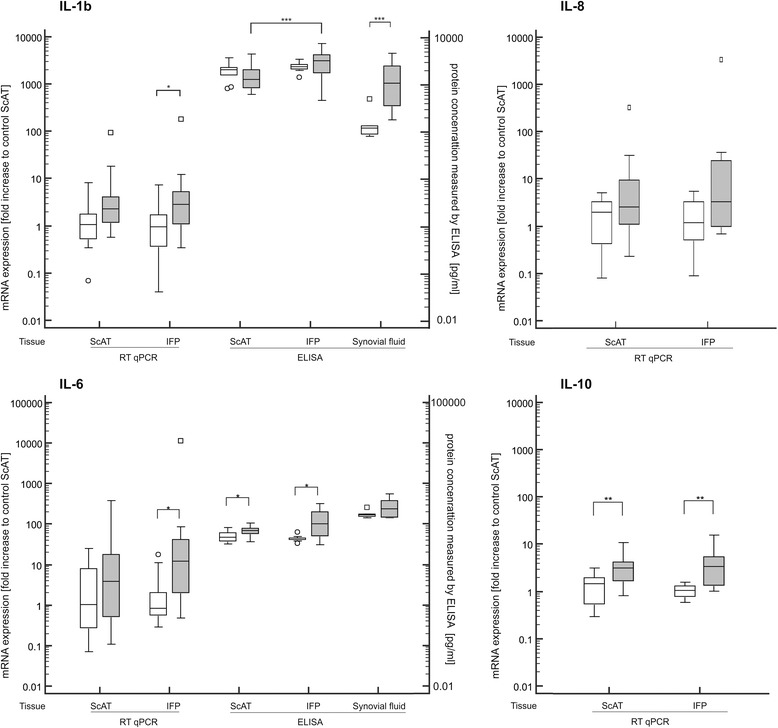
Gene expression and protein release of ScAT and IFP and cytokine concentration in synovial fluid**.** mRNA was quantified by real time RT PCR (RT qPCR). Values of IL-1β, IL-6, IL-8 and IL-10 were normalized to the housekeeping gene (β-actin) and presented as the fold increase compared to control ScAT on the left y-axis. Protein concentration of IL-1β and IL-6 secreted by ScAT and IFP as well as in synovial fluid were measured by ELISA and presented as pg/ mL on the right y-axis. * denotes statistically significant differences with *p* < 0.05, ** *p* < 0.01, and *** *p* < 0.001. White boxes demonstrate control group and grey boxes samples from dogs with CCLD

**Fig. 3:**
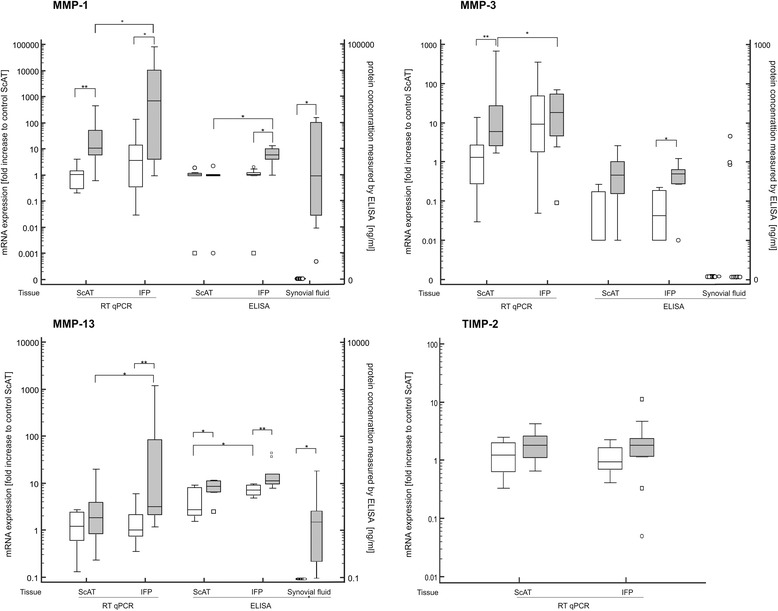
Gene expression and protein release of ScAT and IFP and cytokine concentration in synovial fluid. mRNA was quantified by real time RT PCR (RT qPCR). Values for MMP-1, MMP-3, MMP-13 and TIMP-2 were normalized to the housekeeping gene (β-actin) and presented as the fold increase compared to control ScAT on the left y-axis. Protein concentration of MMP-1, MMP-3 and MMP-13 secreted by ScAT and IFP as well as in synovial fluid were measured by ELISA and presented as ng/ ml on the right y-axis. * denotes statistically significant differences with *p* < 0.05 and ** *p* < 0.01. White boxes demonstrate control group and grey boxes samples from dogs with CCLD

**Fig. 4:**
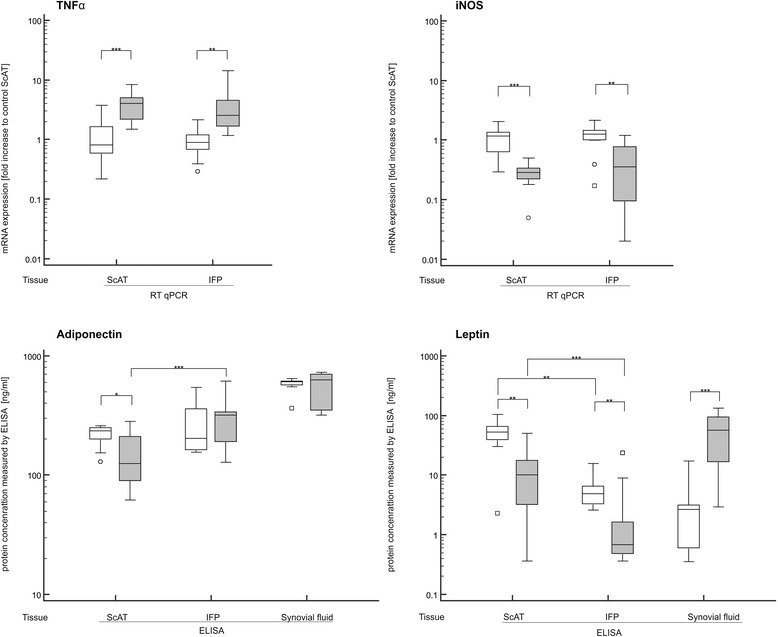
Gene expression and protein release of ScAT and IFP and cytokine concentration in synovial fluid. mRNA was quantified by real time RT PCR (RT qPCR). Values for TNFα and iNOS were normalized to the housekeeping gene (β-actin) and presented as the fold increase compared to control ScAT (upper 2 boxplots). Concentration of adiponectin and leptin secreted by ScAT and IFP as well as in synovial fluid were measured by ELISA and presented as ng/ ml (lower 2 boxplots). * denotes statistically significant differences with *p* < 0.05, ** *p* < 0.01, and *** *p* < 0.001. White boxes demonstrate control group and grey boxes samples from dogs with CCLD

Gene expressions are presented for IL-1β, IL-6, IL-8 and IL-10 in Fig. 2; for MMP-1, MMP-3, MMP-13 and TIMP-2 in Fig. 3 and for iNOS and TNFα in Fig. 4. Comparisons were made to internal paired samples of the thigh ScAT and corresponding tissues of control dogs. The RT-qPCR revealed that genes of various factors, in particular IL-1β (*p* = 0.02), IL-6 (*p* = 0.03), IL-10 (*p* = 0.006), TNFα (*p* = 0.002), MMP-1 (*p* = 0.004) and also MMP-13 (*p* = 0.005) were more activated in the IFP of diseased dogs compared to the IFP of healthy control dogs. Furthermore, the corresponding ScAT of dogs with CCLD showed an increase in quantity of IL-10 (*p* = 0.006) and TNFα (*p* = 0.0005) when compared to control ScAT. Quantity of MMP-3 gene expression was only higher in ScAT of dogs with CCLD than in ScAT of control dogs. All tissue samples expressed MMP-1, which was the most abundantly expressed factor, particularly in IFP from diseased dogs, with a value more than 1500-fold higher than in ScAT of control dogs and more than 700-fold higher than in IFP of control dogs. Moreover, when comparing adipose tissue sources within groups, we found a statistically significant increase in gene expression of MMP-1 (*p* = 0.01), MMP-3 (*p* = 0.03), and MMP-13 (*p* = 0.04) in IFP compared to paired ScAT (see Fig. 3) within the CCLD group. In contrast, gene expression of iNOS was statistically significantly decreased in both tissues (ScAT, *p* = 0.0001; IFP, *p* = 0.008) of the CCLD group compared to the control group. No significant differences were found within or between groups for gene expression of IL-8 and TIMP-2.

### Adipokine, cytokine and MMP secretion

The quantity of selected adipokines, interleukins, and MMPs was measured in the supernatant of IFP- and ScAT-conditioned medium as well as in paired synovial fluid (Figs. 2, 3 and 4). The adipokines adiponectin and leptin were detected in all samples. While no differences of adiponectin secretion were detected within the control group, a statistically significant difference was found in dogs with CCLD. Here, IFP of the involved joints showed higher quantities of adiponectin secreted compared to the paired ScAT (*p* = 0.0001). Interestingly, there was a statistically significant difference in leptin secretion, this being lower in both tissues from diseased dogs compared to controls (*p* < 0.003). Furthermore, a concurrent, statistically significant lower level of secretion in the IFP (*p* < 0.01) was also exhibited in comparison to the ScAT.

Analyzing degrading enzymes, the most obvious differences were detected for MMP-1, similar to the RT-qPCR results. The highest secretion of MMP-1 was detected in IFP from diseased dogs, this being statistically significantly higher compared to paired ScAT (*p* = 0.02) as well as to IFP and ScAT from control dogs (*p* < 0.004). Fat-conditioned media from CCLD dogs of both, IFP and ScAT, contained statistically significantly higher amounts of MMP-13 compared to healthy control dogs. Regarding MMP-3, the concentration was below the detection limit for all control samples. In diseased dogs, only half of the samples yielded measurable values.

Results of interleukin secretion into fat-conditioned media showed that IL-6 was statistically significantly elevated in IFP and ScAT from CCLD dogs compared to control dogs. Moreover, there was a statistically significant higher secretion of IL-1β in the IFP compared to the ScAT (*p* = 0.0002), which was only detected within the CCLD group.

In the synovial fluid from dogs with CCLD, statistically significant higher concentrations of IL-1β, MMP-1 and MMP-13 as well as leptin were detected compared to the control synovial fluid. The concentration of leptin was 20-fold higher than in control synovial fluid (*p* = 0.0002) and on average 70-fold higher compared to the paired IFP (*p* < 0.0001). In the control synovial fluid, MMP-1 and MMP-13 were below the detection limit, while in synovial fluid of dogs suffering from CCLD, six out of eight dogs showed a statistically significant increase (*p* < 0.01) in these two matrix degrading enzymes. MMP-3 was only detected in three samples obtained from dogs with CCLD.

Statistically significant increases were found with progressive duration of lameness in the levels of IL-1β (rs = 0.78, *p* < 0.0001, 95% CI 0.55–0.9), MMP-1 (rs = 0.73, *p* < 0.001, 95% CI 0.38–0.89) and MMP-13 (rs = 0.84, *p* < 0.0001, 95% CI 0.61–0.94). No significant relationships were found between clustered BCS, medication or synovitis and the various inflammatory parameters analyzed.

## Discussion

Today, it is generally accepted that CCLD exhibits a complexity of biomechanical and biological events. Several studies demonstrated that modulatory changes which take place in the synovial lining, in the ligament as well as in the cartilage are initiated through mutual pro-inflammatory cytokines and matrix degrading enzymes, which are subsequently dispersed by synovial fluid [5, 29]. The IFP is an additional intra-articular tissue. To date, in veterinary medicine, in comparison to human medicine, there has been significantly less research performed to investigate the role of IFP in progression of joint pathologies.

In the present study, we were able to demonstrate that the IFP exhibits a pro-inflammatory pattern during CCLD. The activated state of the IFP was apparent through an increased gene expression and/or release of pro-inflammatory cytokines like IL-1β and IL-6, and destructive enzymes like MMP-1 and MMP-13 as well as adipokines such as adiponectin and leptin. Immune cells present in the adipose tissue could be responsible for most of the production and release of inflammatory mediators, except for leptin and adiponectin, which are mainly secreted by adipocytes [30]. In order to gain insight into the possible cellular origin of the secreted inflammatory mediators, we characterized the cell types present in the IFP and ScAT. Our results revealed substantial differences in the immune cell composition between diseased and control dogs. An altered immune cell composition could subsequently explain the higher secreting activity of inflammatory mediators within the IFP in CCLD. The predominant cell types in the IFP which originated from dogs with CCLD were T cells and macrophages. This did not apply to paired ScAT or the corresponding adipose tissue from control dogs which both showed no increased level of these cell types.

Both cell types can generate an enormous spectrum of cytokines including IL-1β, IL-6 and TNFα [31–33]. Moreover, macrophages are suggested to be the major regulator of the production of MMP-1 and MMP-3 [34]. The different cell composition could therefore contribute to the higher release of inflammatory mediators by the IFP in CCLD. Studies analyzing the cell composition of IFP from humans with primary OA showed that macrophages followed by T cells were the most abundant immune cells in this tissue [19]. Although T cell counts were lower than in the subcutaneous adipose tissue, they had a predominant inflammatory phenotype. Various human studies have demonstrated that the type and relative abundance of immune cells are variable and depend on both the type of adipose tissue investigated and the adiposity of the individual [35–38]. Human IFP obtained from patients with CCLD had more classical inflammatory macrophages than IFP from patients with primary OA [37]. In contrast, no correlation between the presence of immune cells and BCS has been obtained in our study. However, the number of animals included in our study resulted in small BCS subgroup size with low statistical power.

Immune cell infiltration is postulated to be a major source of pro-inflammatory mediators perpetuating the inflammatory response in adjacent tissue by paracrine and autocrine effects. Studies looking at synovial changes in various stages of canine OA have found a predominance of T- and B-cells and macrophages [39–41].

Infiltrating immune cells are believed to interact with the resident adipocytes through secretion of cytokines, which leads to reciprocal modulation and change of the inflammatory character of the adipose tissue [18, 42]. Our study determined the level of expression of several inflammatory mediators in ScAT and IFP as well as their levels of proteins secreted into fat-conditioned media. One major finding of our study was that IFP obtained from dogs with CCLD demonstrated higher gene expression levels of IL-1β, IL-6 and TNFα as well as of MMP-1 and MMP-13 compared to both adipose tissues from control dogs. Furthermore, within the CCLD group a significant higher expression of MMP-1, MMP-3 and MMP-13 was detected in the IFP when compared to the paired ScAT. To our knowledge, there is only one publication that investigated the expression of interleukins among other joint tissues in the canine IFP [43]. In contrast to our results, they were not able to show a statistically significant difference in the gene expression of IL-1β and IL-6 between six IFP samples from dogs with CCLD and five healthy control IFP samples. More data is available for human IFP. Investigations of the IFP in human OA joints revealed a similar expression of inflammatory genes as we identified in the IFP of canine CCLD stifle joints [44, 45]. Moreover, human studies also showed an increase protein secretion for several cytokines [45, 46], similar to the significantly increased secretory activity of IL-1β and MMP-1 in the IFP of diseased dogs. The gene expression of iNOS was only significantly downregulated in the IFP of CCLD dogs compared to their healthy control equivalents. Activation of the iNOS system was detected in various joint tissues from dogs suffering from CCLD, suggesting that iNOS is regulated locally and tissue specifically [47].

As the role of adipokines in joint diseases has not been the subject of much investigation in veterinary medicine, we shall address adiponectin and leptin in more detail. A study investigating the inflammatory characteristics of human IFP in knee OA and obesity demonstrated an overproduction of IL-6, which was associated with a decrease of local leptin secretion and an increase in adiponectin secretion [48]. These findings are consistent with our results in dogs with CCLD. In contrast to its protective role against obesity, local adiponectin might act as a pro-inflammatory agent and contribute to matrix degradation in joint tissues [49]. Adiponectin was originally thought to be produced mainly by adipose tissue, meanwhile expression can be induced in synoviocytes as well as in chondrocytes [48, 50]. Because the interaction of articular and periarticular tissues is at least in parts believed to be transmitted through the synovial fluid, we also compared these mediators in the synovial fluid. The concentration of adiponectin in synovial fluid was higher than that secreted by IFP and levels were not different between healthy control and diseased stifle joints, emphasizing that synovial adiponectin is regulated by more than the IFP. Up to date, its role in the pathogenesis of OA is not completely elucidated. There is some evidence that adiponectin affects IL-1β, IL-6, MMP-1, MMP-3, MMP-13 and NO secretion and suppresses mature macrophage functions such as phagocytosis [51, 52].

Findings regarding leptin are inconsistent in human literature. While some investigators propose a pro-inflammatory role of leptin in the regulation of cartilage metabolism via activation of MMPs [42, 53, 54] others have identified a decrease of locally produced leptin by the IFP [48] in obese OA patients. Concentration of plasma leptin is positively correlated with increased storage of subcutaneous adipose tissue [55]. However, the majority of studies showed that the release of leptin and adiponectin was independent of body mass and gender [56], which is in concordance with our results. Interestingly, we found elevated levels of synovial leptin (in average 70-fold) compared to the IFP production of dogs with CCLD, suggesting that other local tissue must release leptin into the synovial fluid. Recent findings indicate that deteriorated articular cartilage and synoviocytes are another source of adiponectin and leptin within the joint [57].

A limitation of the present study is the fact that we measured the mediators only in IFP, ScAT and synovial fluid. Thus, although we were able to characterize the inflammatory potential of the adipose tissue, it was not possible to differentiate the origin of mediator production, thereby imposing a restriction on the possibilities for understanding the pathophysiological pathways involved. Additional paired samples of the synovial membrane, ligaments as well as cartilage would have offered a more comprehensive picture and thereby provided assistance in the identification of the pathways. Furthermore, grading of the clinical severity of CCLD and BCS is nonetheless subjective and this may have introduced some insensitive measure bias. We believe that bias and the limited number of samples may be responsible for some of the wide variability associated with gene expression and protein production between the BCS subgroups. The used ELISA for MMP-3 in which measured values were recorded below detection limit could also be considered a limitation. However, various studies were not able to detect MMPs in synovial fluid [14, 58]. One reason could be that MMP-3 strongly expressed in early OA and down regulated in late phase which might establish the observed differences [59]. Therefore, the use of this kit was considered a minor limitation, but may have affected the results associated with MMP-3. In contrast to similar studies in human medicine, the ability in animal studies to obtain control tissue from a healthy control group poses a major advantage.

## Conclusions

In conclusion, as reported in similar findings in human medicine, the results of the present study indicate that the IFP has to be considered as a potential factor in the pathogenesis of CCLD. We showed that the IFP might have specific inflammatory phenotypic features independent from the general phenotype found in obesity. Because dogs are often used as models in human medicine, the present and similar studies could be regarded with great interest there to gain further insight into the processes involved. Nevertheless, it would be very interesting to obtain specimens of adjacent joint tissues. Future investigations must be carried out in order to estimate the exact impact of this immunomodulatory tissue within the stifle joint on causing or driving this particular disease.

## Abbreviations

BCS: Body Condition Score
CCL: Canine Cruciate Ligament
CCLD: Canine Cruciate Ligament Disease
CD: Cluster of Differentiation
CI: Confidential Interval
DMEM: Dulbecco’s Modified Eagle’s Medium
DTT: Dithiothreitol
ELISA: Enzyme-linked Immunosorbent Assay
FACS: Fluorescence-activated Cell Sorting
IFP: Infrapatellar Fat Pad
IL: Interleukin
iNOS: inducible Nitric Oxide Synthase
MMP: Matrix Metalloproteinases
mRNA: messenger Ribonucleic Acid
NO: Nitric Oxide
OA: Osteoarthritis
*p*: *P*-value
PBS: Phosphate Buffered Saline
RNA: Ribonucleic Acid
rs: Spearman’s Correlation Coefficient
RT-qPCR: Quantitative Real-time Reverse Transcriptase Polymerase Chain Reaction
ScAT: Subcutaneous Adipose Tissue
TIMP: Tissue Inhibitor of Metalloproteinases
TNF: Tumor Necrosis Factor

## References

[1] Bennett D, Tennant B, Lewis DG, Baughan J, May C, Carter SA (1988). Reappraisal of anterior cruciate ligament disease in the dog. J Small Anim Pract.

[2] Johnson JM, Johnson AL (1993). Cranial cruciate ligament rupture. Pathogenesis, diagnosis, and postoperative rehabilitation. Vet Clin N am-Small.

[3] Innes JF, Costello M, Barr FJ, Rudorf H, Barr AR (2004). Radiographic progression of osteoarthritis of the canine stifle joint: a prospective study. Vet Radiol Ultrasoun.

[4] Rayward RM, Thomson DG, Davies JV, Innes JF, Whitelock RG (2004). Progression of osteoarthritis following TPLO surgery: a prospective radiographic study of 40 dogs. J Small Anim Pract.

[5] Comerford EJ, Smith K, Hayashi K (2011). Update on the aetiopathogenesis of canine cranial cruciate ligament disease. Vet Comp Orthopaed.

[6] Doom M, de Bruin T, de Rooster H, van Bree H, Cox E (2008). Immunopathological mechanisms in dogs with rupture of the cranial cruciate ligament. Vet Immunol Immunop.

[7] Erne JB, Goring RL, Kennedy FA, Schoenborn WC (2009). Prevalence of lymphoplasmacytic synovitis in dogs with naturally occurring cranial cruciate ligament rupture. J Am Vet Med Assoc.

[8] Muir P, Schamberger GM, Manley PA, Hao Z (2005). Localization of cathepsin K and tartrate-resistant acid phosphatase in synovium and cranial cruciate ligament in dogs with cruciate disease. Vet Surg.

[9] Boland L, Danger R, Cabon Q, Rabillard M, Brouard S, Bouvy B, Gauthier O (2014). MMP-2 as an early synovial biomarker for cranial cruciate ligament disease in dogs. Veterinary and comparative orthopaedics and traumatology. Vet Comp Orthopaed..

[10] Fujita Y, Hara Y, Nezu Y, Schulz KS, Tagawa M (2006). Proinflammatory cytokine activities, matrix metalloproteinase-3 activity, and sulfated glycosaminoglycan content in synovial fluid of dogs with naturally acquired cranial cruciate ligament rupture. Vet Surg.

[11] Hay CW, Chu Q, Budsberg SC, Clayton MK, Johnson KA (1997). Synovial fluid interleukin 6, tumor necrosis factor, and nitric oxide values in dogs with osteoarthritis secondary to cranial cruciate ligament rupture. Am J Vet Res.

[12] El-Hadi M, Charavaryamath C, Aebischer A, Smith CW, Shmon C, Singh B (2012). Expression of interleukin-8 and intercellular cell adhesion molecule-1 in the synovial membrane and cranial cruciate ligament of dogs after rupture of the ligament. Can J Vet Res.

[13] Muir P, Danova NA, Argyle DJ, Manley PA, Hao Z (2005). Collagenolytic protease expression in cranial cruciate ligament and stifle synovial fluid in dogs with cranial cruciate ligament rupture. Vet Surg.

[14] Breshears LA, Cook JL, Stoker AM, Fox DB (2010). Detection and evaluation of matrix metalloproteinases involved in cruciate ligament disease in dogs using multiplex bead technology. Vet Surg.

[15] Mac CM (1950). The movements of bones and joints; the synovial fluid and its assistants. J Bone Joint Surg Br.

[16] Dumond H, Presle N, Terlain B, Mainard D, Loeuille D, Netter P, Pottie P (2003). Evidence for a key role of leptin in osteoarthritis. Arthritis Rheum.

[17] Ushiyama T, Chano T, Inoue K, Matsusue Y (2003). Cytokine production in the infrapatellar fat pad: another source of cytokines in knee synovial fluids. Ann Rheum Dis.

[18] Clockaerts S, Bastiaansen-Jenniskens YM, Runhaar J, Van Osch GJ, Van Offel JF, Verhaar JA, De Clerck LS, Somville J (2010). The infrapatellar fat pad should be considered as an active osteoarthritic joint tissue: a narrative review. Osteoarthr. Cartilage..

[19] Klein-Wieringa IR, Kloppenburg M, Bastiaansen-Jenniskens YM, Yusuf E, Kwekkeboom JC, El-Bannoudi H, Nelissen RG, Zuurmond A, Stojanovic-Susulic V, Van Osch GJ (2011). The infrapatellar fat pad of patients with osteoarthritis has an inflammatory phenotype. Ann Rheum Dis.

[20] Anderson EK, Gutierrez DA, Hasty AH (2010). Adipose tissue recruitment of leukocytes. Curr Opin Lipidol.

[21] Issa RI, Griffin TM (2012). Pathobiology of obesity and osteoarthritis: integrating biomechanics and inflammation. Pathobiol Aging Age Relat Dis.

[22] Ioan-Facsinay A, Kwekkeboom JC, Westhoff S, Giera M, Rombouts Y, van Harmelen V, Huizinga TW, Deelder A, Kloppenburg M, Toes RE (2013). Adipocyte-derived lipids modulate CD4+ T-cell function. Eur J Immunol.

[23] Kershaw EE, Flier JS (2004). Adipose tissue as an endocrine organ. J Clin Endocr Metab.

[24] Fantuzzi G (2005). Adipose tissue, adipokines, and inflammation. J Allergy Clin Immun.

[25] Witonski D, Wagrowska-Danilewicz M, Keska R, Raczynska-Witonska G, Stasikowska-Kanicka O (2010). Increased interleukin 6 and tumour necrosis factor alpha expression in the infrapatellar fat pad of the knee joint with the anterior knee pain syndrome: a preliminary report. Pol J Pathol.

[26] Ioan-Facsinay A, Kloppenburg M (2013). An emerging player in knee osteoarthritis: the infrapatellar fat pad. Arthritis Res Ther.

[27] Development LDP (1997). Validation of a body condition score system for dogs. Canine Pract.

[28] Livak KJ, Schmittgen TD (2001). Analysis of relative gene expression data using real-time quantitative PCR and the 2(−Delta Delta C(T)) method. Methods.

[29] Muir P, Kelly JL, Marvel SJ, Heinrich DA, Schaefer SL, Manley PA, Tewari K, Singh A, Suresh M, Hao Z (2011). Lymphocyte populations in joint tissues from dogs with inflammatory stifle arthritis and associated degenerative cranial cruciate ligament rupture. Vet Surg.

[30] Fain JN (2006). Release of interleukins and other inflammatory cytokines by human adipose tissue is enhanced in obesity and primarily due to the nonfat cells. Vitam Horm.

[31] Bondeson J, Wainwright SD, Lauder S, Amos N, Hughes CE (2006). The role of synovial macrophages and macrophage-produced cytokines in driving aggrecanases, matrix metalloproteinases, and other destructive and inflammatory responses in osteoarthritis. Arthritis Res Ther..

[32] Burger D, Dayer JM (2002). The role of human T-lymphocyte-monocyte contact in inflammation and tissue destruction. Arthritis Res.

[33] Ishii H, Tanaka H, Katoh K, Nakamura H, Nagashima M, Yoshino S (2002). Characterization of infiltrating T cells and Th1/Th2-type cytokines in the synovium of patients with osteoarthritis. Osteoarthr. Cartilage..

[34] Kinne RW, Stuhlmuller B, Burmester GR (2007). Cells of the synovium in rheumatoid arthritis. Macrophages Athritis Res Ther.

[35] Caspar-Bauguil S, Cousin B, Galinier A, Segafredo C, Nibbelink M, Andre A, Casteilla L, Penicaud L (2005). Adipose tissues as an ancestral immune organ: site-specific change in obesity. FEBS Lett.

[36] Kontny E, Prochorec-Sobieszek M (2013). Articular adipose tissue resident macrophages in rheumatoid arthritis patients: potential contribution to local abnormalities. Rheumatology.

[37] Bastiaansen-Jenniskens YM, Clockaerts S, Feijt C, Zuurmond AM, Stojanovic-Susulic V, Bridts C, de Clerck L, DeGroot J, Verhaar JA, Kloppenburg M (2012). Infrapatellar fat pad of patients with end-stage osteoarthritis inhibits catabolic mediators in cartilage. Ann Rheum Dis.

[38] Clements KM, Ball AD, Jones HB, Brinckmann S, Read SJ, Cellular MF (2009). Histopathological changes in the infrapatellar fat pad in the monoiodoacetate model of osteoarthritis pain. Osteoarthr. Cartilage.

[39] Galloway RH, Lester SJ (1995). Histopathological evaluation of canine stifle joint synovial membrane collected at the time of repair of cranial cruciate ligament rupture. J Am Anim Hosp Assoc.

[40] Faldyna M, Zatloukal J, Leva L, Kohout P (2004). Lymphocyte subsetzs in stifle joint synovial fluid of dogs with spontaneous rupture of the cranial cruciate ligament. Acta Vet Brno.

[41] Lemburg AK, Meyer-Lindenberg A, Hewicker-Trautwein M (2004). Immunohistochemical characterization of inflammatory cell populations and adhesion molecule expression in synovial membranes from dogs with spontaneous cranial cruciate ligament rupture. Vet Immunol Immunop..

[42] Gandhi R, Takahashi M, Virtanen C, Syed K, Davey JR, Mahomed NN (2011). Microarray analysis of the infrapatellar fat pad in knee osteoarthritis: relationship with joint inflammation. J Rheumatol.

[43] Maccoux LJ, Salway F, Day PJ, Clements DN (2007). Expression profiling of select cytokines in canine osteoarthritis tissues. Vet Immunol Immunop..

[44] Clockaerts S, Bastiaansen-Jenniskens YM, Feijt C, De Clerck L, Verhaar JA, Zuurmond AM, Stojanovic-Susulic V, Somville J, Kloppenburg M, van Osch GJ (2012). Cytokine production by infrapatellar fat pad can be stimulated by interleukin 1beta and inhibited by peroxisome proliferator activated receptor alpha agonist. Ann Rheum Dis.

[45] de Jong AJ, Klein-Wieringa IR, Kwekkeboom JC, Toes REM, Kloppenburg M, Ioan-Facsinay A. Inflammatory features of infrapatellar fat pad in rheumatoid arthritis versus osteoarthritis reveal mostly qualitative differences. Ann Rheum Dis. 2017;211673 10.1136/annrheumdis-2017-211673 28780513

[46] Eymard F, Pigenet A, Citadelle D, Flouzat-Lachaniette CH, Poignard A, Benelli C, Berenbaum F, Chevalier X, Houard X (2014). Induction of an inflammatory and prodegradative phenotype in autologous fibroblast-like synoviocytes by the infrapatellar fat pad from patients with knee osteoarthritis. Arthritis Rheumatol.

[47] Louis E, Remer KA, Doherr MG, Neumann U, Jungi T, Schawalder P, Spreng D (1723). Nitric oxide and metalloproteinases in canine articular ligaments: a comparison between the cranial cruciate, the medial genual collateral and the femoral head ligament. Vet J.

[48] Distel E, Cadoudal T, Durant S, Poignard A, Chevalier X, Benelli C (2009). The infrapatellar fat pad in knee osteoarthritis: an important source of interleukin-6 and its soluble receptor. Arthritis Rheum.

[49] Lago R, Gomez R, Otero M, Lago F, Gallego R, Dieguez C, Gomez-Reino JJ, Gualillo OA (2008). New player in cartilage homeostasis: adiponectin induces nitric oxide synthase type II and pro-inflammatory cytokines in chondrocytes. Osteoarthr. Cartilage..

[50] Presle N, Pottie P, Dumond H, Guillaume C, Lapicque F, Pallu S, Mainard D, Netter P, Terlain B (2006). Differential distribution of adipokines between serum and synovial fluid in patients with osteoarthritis. Contribution of joint tissues to their articular production. Osteoarthr. Cartilage..

[51] Kang EH, Lee YJ, Kim TK, Chang CB, Chung JH, Shin K, Lee EY, Lee EB, Song YW (2010). Adiponectin is a potential catabolic mediator in osteoarthritis cartilage. Arthritis Res Ther..

[52] Hu PF, Bao JP, Wu LD (2011). The emerging role of adipokines in osteoarthritis: a narrative review. Mol Biol Rep.

[53] Otero M, Lago R, Lago F, Reino JJ, Gualillo O (2005). Signalling pathway involved in nitric oxide synthase type II activation in chondrocytes: synergistic effect of leptin with interleukin-1. Arthritis Res Ther..

[54] Hui W, Litherland GJ, Elias MS, Kitson GI, Cawston TE, Rowan AD, Young DA (2012). Leptin produced by joint white adipose tissue induces cartilage degradation via upregulation and activation of matrix metalloproteinases. Ann Rheum Dis.

[55] Saad MF, Damani S, Gingerich RL, Riad-Gabriel MG, Khan A, Boyadjian R, Jinagouda SD (1997). el-Tawil K, rude RK, Kamdar V. Sexual dimorphism in plasma leptin concentration. J Clin Endocr Metab..

[56] Gross JB, Guillaume C, Gegout-Pottie P, Reboul P, Jouzeau JY, Mainard D, Presle N (2017). The infrapatellar fat pad induces inflammatory and degradative effects in articular cells but not through leptin or adiponectin. Clin Exp Rheumatol.

[57] Koskinen A, Juslin S, Nieminen R, Moilanen T, Vuolteenaho K, Moilanen E (2011). Adiponectin associates with markers of cartilage degradation in osteoarthritis and induces production of proinflammatory and catabolic factors through mitogen-activated protein kinase pathways. Arthritis Res Ther..

[58] Rabillard M, Danger R, Doran IP, Niebauer GW, Brouard S, Gauthier O (2012). Matrix metalloproteinase activity in stifle synovial fluid of cranial cruciate ligament deficient dogs and effect of postoperative doxycycline treatment. Vet J.

[59] Aigner T, Zien A, Gehrsitz A, Gebhard PM, McKenna L (2001). Anabolic and catabolic gene expression pattern analysis in normal versus osteoarthritic cartilage using complementary DNA-array technology. Arthritis Rheum.

[60] de Bruin T, de Rooster H, Bosmans T, Duchateau L, van Bree H, Gielen I. Radiographic assessment of the progression of osteoarthrosis in the contralateral stifle joint of dogs with a ruptured cranial cruciate ligament. Vet Rec 2007;16122:745–750. 10.1136/vr.161.22.745 18056011

[61] Little JP, Bleedorn JA, Sutherland BJ, Sullivan R, Kalscheur VL, Ramaker MA, Schaefer SL, Hao Z, Muir P (2014). Arthroscopic assessment of stifle synovitis in dogs with cranial cruciate ligament rupture. PLoS One.

